# Ultraviolet-C Photoresponsivity Using Fabricated TiO_2_ Thin Films and Transimpedance-Amplifier-Based Test Setup

**DOI:** 10.3390/s22218176

**Published:** 2022-10-25

**Authors:** Marilou Cadatal-Raduban, Jade Pope, Jiří Olejníček, Michal Kohout, John A. Harrison, S. M. Rezaul Hasan

**Affiliations:** 1 School of Natural Sciences, Massey University, Auckland 0632, New Zealand; 2 Institute of Laser Engineering, Osaka University, 2-6 Yamadaoka, Suita 565-0871, Osaka, Japan; 3 Department of Low Temperature Plasma, Institute of Physics of the Czech Academy of Sciences, Na Slovance 1999/2, 182 21 Prague 8, Czech Republic; 4 Center for Research in Analog and VLSI Microsystem Design, Massey University, Auckland 0632, New Zealand

**Keywords:** titanium dioxide, thin film, ultraviolet, UV-C, photoconductive detector, sensor, transimpedance amplifier

## Abstract

We report on fabricated titanium dioxide (TiO_2_) thin films along with a transimpedance amplifier (TIA) test setup as a photoconductivity detector (sensor) in the ultraviolet-C (UV-C) wavelength region, particularly at 260 nm. TiO_2_ thin films deposited on high-resistivity undoped silicon-substrate at thicknesses of 100, 500, and 1000 nm exhibited photoresponsivities of 81.6, 55.6, and 19.6 mA/W, respectively, at 30 V bias voltage. Despite improvements in the crystallinity of the thicker films, the decrease in photocurrent, photoconductivity, photoconductance, and photoresponsivity in thicker films is attributed to an increased number of defects. Varying the thickness of the film can, however, be leveraged to control the wavelength response of the detector. Future development of a chip-based portable UV-C detector using TiO_2_ thin films will open new opportunities for a wide range of applications.

## 1. Introduction

Ultraviolet radiation (UV), broadly used to refer to the wavelength range from 100 nm to 400 nm, has widespread and important scientific, industrial, and medical applications including spectroscopy, photochemical processing, disinfection, and sterilization. UV-C (100–280 nm wavelength) provides an environmentally friendly and chemical-free means for sterilization because of its germicidal effects on a broad spectrum of micro-organisms [1,2,3,4,5]. The detrimental effect to the microbial cells is due to the critical damage inflicted to the microbes’ genetic material (RNA, DNA, and proteins) by the absorbed UV-C photons. The micro-organism is most vulnerable to photons with a 260 nm wavelength, where the relative susceptibility of RNA and DNA also peaks [6]. Recent works have particularly shown the efficacy of UV-C in completely inactivating corona viruses, including SARS-CoV-2, on surfaces or aerosols [7,8,9,10]. Research on portable light sources emitting UV-C wavelengths has seen tremendous growth, especially due to demands for sterilizing surfaces [11,12]. It is expected that development of portable UV-C detectors would follow suit.

Photoconductive detectors made with semiconductors are most promising for developing portable chip-based detectors because of their efficiency, reliability, and simplicity [13]. The wavelength detection limit of such detectors is dictated by the band gap of the semiconductor material, without having to use optical filters. Furthermore, the band gap can be tuned by controlling the composition ratio [14] and the fabrication parameters [15,16]. Zinc oxide (ZnO) [17], gallium nitride (GaN) [18], aluminum nitride (AlN) [19,20], and boron nitride (BN) [21,22,23,24,25] are semiconductor materials that have been reported as UV photoconductive detectors, each with their own advantages and disadvantages. Diamond has also been reported for UV detection, however, it is a less attractive option due to the high production cost and requirement for low defect concentration as well as the difficulty of incorporating n-type and p-type dopants [26,27,28]. Additionally, the band gap of diamond is not tunable. In this paper, we explore the photoconductivity of titanium dioxide (TiO_2_) thin films in the UV-C wavelength region, especially with 260 nm wavelength excitation. Mostly known for its UV photocatalytic activity, TiO_2_ has excellent chemical, physical, and optical properties including being a wide band-gap semiconductor [29,30]. Compared with ZnO, GaN, AlN, and BN, TiO_2_ is relatively easier and cheaper to fabricate. The wide band gap of TiO_2_ allows detection of UV-C radiation without having to use filters to block out unwanted visible radiation. In conjunction with the UV-C thin-film photoconductive material, a chip-based readout would be necessary when developing a portable detector. Previously, we have reported a Floating Gate Radiation Field Effect Transistor (FGRADFET) sensor, which is a single-chip implementation employing standard single-poly 130 nm CMOS technology [13]. Although the implementation of such a sensor was successful in determining the absorbed dose and dose rate with X-ray excitation, the photoactive material cannot be readily changed from what is commercially available because of the nanometer-sized structure of the channel and gates. In this work, the photoconductivity of TiO_2_ thin-film layers deposited on a high-resistivity undoped silicon (Si) substrate was measured in conjunction with a custom-designed transimpedance amplifier (TIA) chip for future development of a chip-based portable UV-C detector. The effect of film thickness on the photoconductivity of TiO_2_ thin films deposited on the Si substrate is also investigated for the first time. Our results show that the increasing number of defects in thicker films will be an important consideration when developing a portable photoconductive detector for sensing high-energy radiation such as X-rays, where the high penetration depth would require film thicknesses of a few tens of micrometers.

## 2. Materials and Methods

### 2.1. Preparation of TiO_2_ Thin-Film Photoconductive Detector

**Figure 1:**
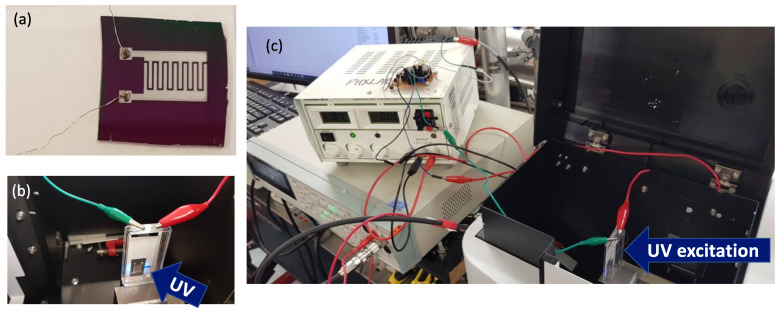
(**a**) Photograph of the TiO_2_ thin-film photoconductive detector. (**b**) Photograph of the detector being illuminated by 260 nm UV-C radiation. (**c**) Photograph of the experimental setup used to measure the photoconductivity of the detectors.

Reactive direct current (DC) magnetron sputtering was used to fabricate the TiO_2_ thin films. Before deposition, the deposition chamber was evacuated to 1 × 10^−3^ Pa base pressure. A pure titanium (99.995% purity) target was used to sputter the films onto a high-resistivity undoped Si substrate. The target has a diameter of 6 inches (15 cm). The Si substrate has a thickness of 0.80 mm, resistivity of 1 kΩ·cm, and an orientation in the (1 0 0) plane. The films were sputtered in reactive oxygen (O_2_) and argon (Ar) atmosphere, maintaining an O_2_: Ar gas mixture ratio of 1:4. This ratio was achieved by keeping a constant flow rate of 5 sccm for O_2_ and 20 sccm for Ar. The total gas pressure was controlled at 1.3 Pa during deposition. A deposition rate of 5 nm/min was achieved by using a power density of 3.4 W/cm^2^, corresponding to an absorbed power of 600 W. To evaluate the effect of thickness on photoconductivity, films with a thickness of 100 nm, 500 nm, and 1000 nm were fabricated. The desired thickness of the deposited film was obtained by adjusting the deposition time. The thickness of the films was also confirmed after deposition using a surface profilometer with an accuracy error under ±10%. To obtain crystalline TiO_2_ with good stoichiometry, the deposited films were annealed at 450 C in air for 8 h. A pair of interdigitated electrodes were then deposited on the surface of the films to fabricate the photoconductive detector shown in Figure 1a. A pure Al (99.99%) target was used to deposit the electrodes through a stainless-steel mask using the same chamber used in depositing the TiO_2_ films. Deposition of the electrode was carried out in Ar atmosphere with an Ar flow rate of 20 sccm and a working pressure of 1 Pa. The length of the electrode is 7.8 mm, and the distance between the electrode pair is 0.2 mm, giving a sensing area of about 75 mm^2^. The thickness of the electrode is 500 nm.

### 2.2. Characterization of the Structural and Optical Properties of the TiO_2_ Thin Films

An Empyrean X-ray diffractometer (XRD) fitted with a Cu Kα source emitting at a wavelength of 0.154 nm was used to evaluate the crystallinity, strain, and crystallite or particle size of the TiO_2_ films. The X-ray diffractometer was operated in grazing incidence geometry with a 2*θ* range of 10 to 90 and a step size of 0.01. The diffraction patterns were analyzed qualitatively using a HighScore Plus 4.0 software package to obtain the peak positions and their corresponding full-width at half-maximum (FWHM) broadening. The strain and the crystallite size for each film was obtained using the Williamson–Hall plot method, whereby the total broadening (FWHM) observed in the XRD spectrum is a combination of the broadening due to the crystallite size ($\beta_{D}$) and due to the strain ($\beta_{\varepsilon }$) according to Equations (1) and (2):

(1) $$\beta_{D}=\frac{K\lambda }{D\cos\theta }$$

also known as the Scherrer formula, where $\beta_{D}$ is the FWHM broadening due to the crystallite size, K is the shape factor that is equal to 0.9, λ is the wavelength of the X-ray source, which is 0.154 nm, D is the crystallite size, and θ is the peak position in radians.

(2) $$\beta_{\varepsilon }=4\varepsilon \tan\theta$$

where $\beta_{\varepsilon }$ is the FWHM broadening due to strain, ε is the strain, and θ is the peak position in radians.

By deconvolving the XRD spectrum into the amorphous phase contribution (broad curves) and crystalline contribution (sharp diffraction peaks) and integrating each of these contributions, the degree of crystallinity for each film can be calculated from the ratio of the crystalline contribution ($A_{c}$) to the amorphous contribution ($A_{a})$ according to

(3) $$\mathrm{crystallinity}=\frac{A_{c}}{A_{c}+A_{a}}\times 100$$

The optical properties of the TiO_2_ films were evaluated using their transmission spectra from 200 nm to 700 nm, which were obtained using a double-beam UV-visible near-infrared spectrophotometer. Since Si is opaque to optical wavelengths, identical films were fabricated on a highly transmitting quartz glass substrate. The deposition of the films on quartz glass were carried out at the same time as the films on Si to ensure the same deposition parameters and thickness. The photoluminescence emission spectra of the films were obtained using a spectrofluorophotometer fitted with a 150 W xenon lamp. The 260 nm UV-C excitation wavelength was selected by a monochromator and was incident on the surface of the TiO_2_ film. Photoluminescence emission spectra from the thin film were measured by scanning the grating of the emission spectrometer after passing through a 280 nm high-pass optical filter. The photomultiplier tube (PMT) output signal was corrected for the variation in wavelength sensitivity of the instrument.

### 2.3. Transimpedance Amplifier-Based Test Setup and Evaluation of Photoconductivity of the TiO_2_ Detectors

**Figure 2:**
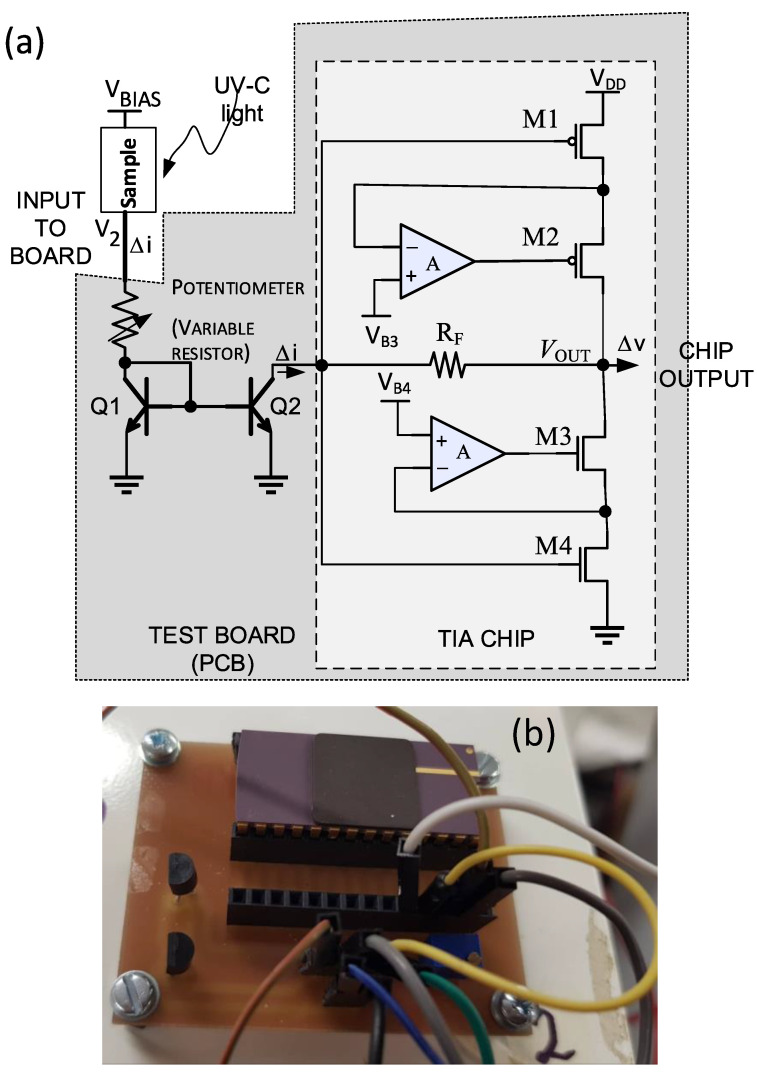
(**a**) Schematic diagram of the TIA chip and test board setup and (**b**) photograph of the fabricated test board with TIA chip.

The photoconductivity of the thin-film photodetector was evaluated using a transimpedance amplifier (TIA)-based test setup. The circuit diagram of the TIA test setup and the photograph of the test board with the TIA chip are shown in the Figure 2a,b, respectively. The breakdown voltage of the discrete bipolar transistor (BC337) used for the current mirror devices Q1 and Q2 is about 45 V, which defines the maximum bias voltage applied to the detector. Therefore, in measuring the dark currents and the photocurrents, a bias voltage of up to 40 V was applied to the TiO_2_ detector. A VDD of 1.8 V was applied to the TIA chip. The resistance of the potentiometer was set to 100 Ω. The chip output and input is biased at 0.9 V. The bias voltage of 0.9 V at the collector of Q2 (TIA input) and the diode connection of Q1 ensures that both Q1 and Q2 operate in the forward-active region, and the current mirror functions accurately. The TIA chip was designed in-house and fabricated through the TSMC 180 nm CMOS Foundry. The TIA has a *g*_m_-boosted inverter configuration with resistor feedback biasing provided by R_F_.

The sensing area of the thin-film detector was illuminated with the 260 nm UV-C emission of a xenon lamp, as shown in Figure 1b. A bias voltage of up to 40 V was applied to one of the aluminum electrodes, and the other electrode was connected to the potentiometer at the test board input as shown in the Figure 2a. As a result, the photo-generated carriers created a drift current due to the applied electric field. A photocurrent was thus generated that is fed to the transimpedance amplifier through the current mirror formed by Q1 and Q2. The output from the TIA was measured using an ultra-high-resistance electrometer. The equipment set-up is shown in the Figure 1c. The dark current was measured using the same measurement setup, except that the detector was kept in the dark by turning off the xenon lamp. The wavelength response of the titanium-dioxide detectors was evaluated by tuning the excitation wavelength from 200 nm to 400 nm, while a 40 V bias voltage is applied to the detector. Since the monochromator’s efficiency and the lamp’s emissivity changes over this wavelength range; the measured photocurrents were corrected for the wavelength dependence of the excitation source.

## 3. Results and Discussion

**Figure 3:**
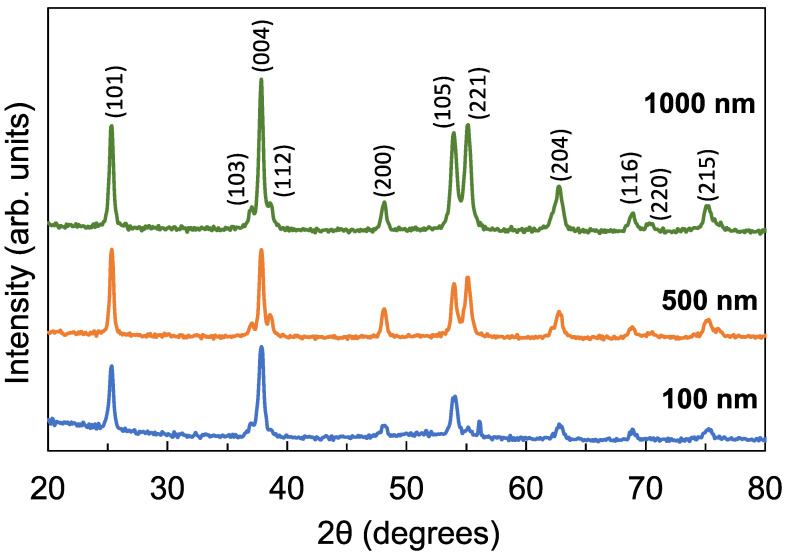
XRD spectra of the TiO_2_ films with film thicknesses of 100 nm, 500 nm, and 1000 nm. All peaks are indexed to the anatase phase of TiO_2_.

Figure 3 shows the XRD spectra of the TiO_2_ films with a thickness of 100 nm, 500 nm, and 1000 nm. All the diffraction peaks observed can be assigned to the anatase phase of TiO_2_. The films have a preferred grain orientation in the (0 0 4) direction at 37.8. While all the films are crystalline, the degree of crystallinity improved from 50% to 81% as the thickness increased. The crystallite size likewise increased from 9.6 nm to 26.6 nm as the thickness increased. On the other hand, the strain decreased from 2.4 × 10^−3^ to 5.2 × 10^−5^ as the thickness increased. These results are summarized in Table 1.

**Figure 4:**
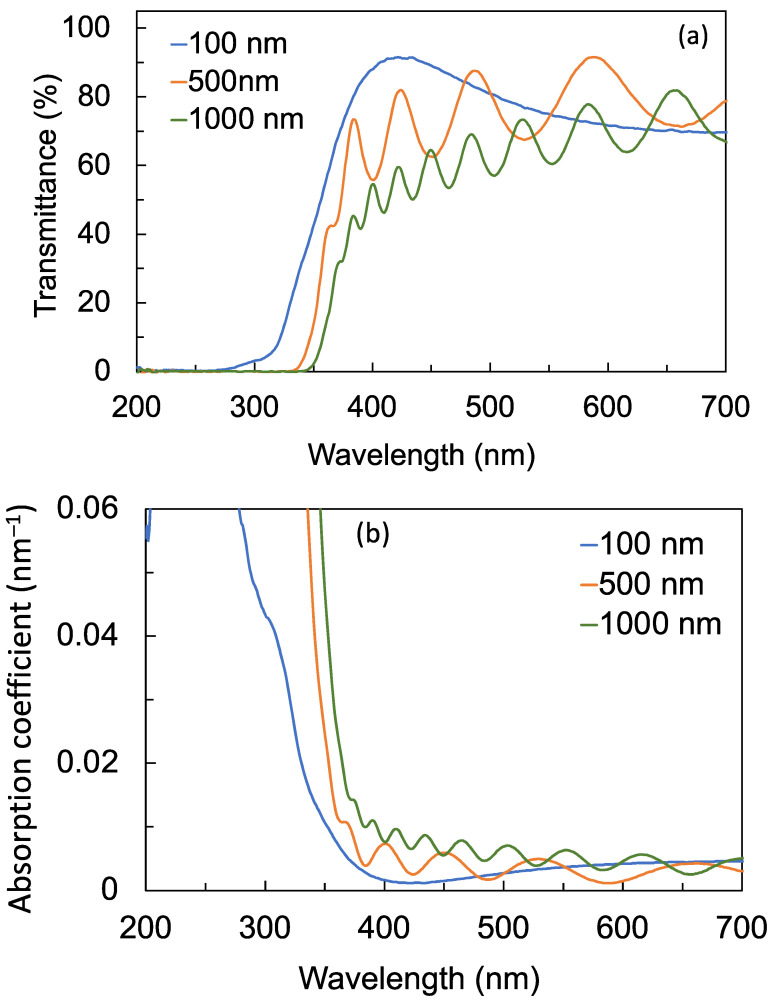
(**a**) Transmission and (**b**) absorption spectra of the TiO_2_ films with film thicknesses of 100 nm, 500 nm, and 1000 nm. The Fabry–Perot fringes observed in the spectra are due to multiple reflections from the substrate.

The transmission and absorption spectra of the films are shown in Figure 4a,b, respectively. The transmission spectra of the films on the substrate were divided by the transmission spectrum of a reference substrate to exclude the contribution of the substrate. The sinusoidal fluctuations in the transmittance curves are Fabry–Perot interference fringes. The films have similar transmittances at the long-wavelength region, but a significant decrease in transmittance at the short-wavelength region between the transmission edge and about 450 nm can be observed. Furthermore, the transmission and absorption edges are shifted to the longer wavelength (red-shifted) as the thickness of the film increased. The band gaps of the films were estimated using Tauc plots obtained from the absorption spectra. The band gap decreased from 3.49 eV to 3.34 eV as the thickness increased, as shown in Table 2. The decrease in band-gap energy is consistent with the observed red shift in the transmission and absorption edges as the thickness increased. The decrease in the band-gap energy and the red shift in the transmission and absorption edges is ascribed to the increase in defect states as the thickness of the film increased, since defect states tend to form just below the conduction band. The most common defects formed in TiO_2_ are oxygen vacancies and trivalent titanium (Ti^3+^) ions. These defects create deep electron traps that are manifested as localized states. It has been estimated that these defect states are located between 0.75 eV to 1.00 eV below the conduction-band minimum [31,32].

**Figure 5:**
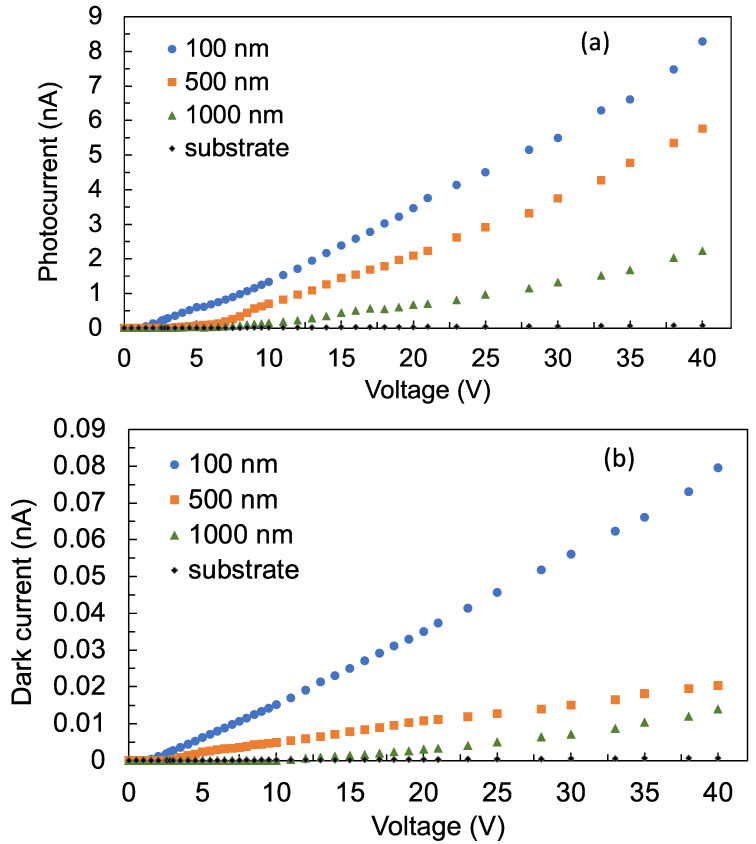
I-V characteristics of the detectors with 100 nm, 500 nm, and 1000 nm film thicknesses (**a**) under 260 nm UV-C illumination and (**b**) under darkness when the light source is turned off.

Figure 5a shows the current voltage (I-V) characteristics of the thin-film photoconductive detectors under 260 nm UV-C illumination, while Figure 5b shows the I-V characteristics under darkness. The photocurrent is observed to be about two orders of magnitude greater than the dark current, indicating that the TiO_2_ films are photoconductive. The detector with the 100 nm film thickness exhibits a linear increase in the photocurrent as the bias voltage is increased. On the other hand, the detectors with the 500 nm and 1000 nm films exhibit a nonlinear increase in the photocurrent at low bias voltages, before eventually displaying a linear increase in the photocurrent from about 10 V bias voltage. The nonlinear increase in photocurrent implies that the mobility of photo-generated carriers is reduced. Reduced mobility is due to the presence of defects in the film. As discussed in the preceding paragraph, the number of defects increased as the thickness of the film increased. These defects also serve as trapping centers for the photo-generated carriers. Consequently, the carrier density in the detectors with 500 nm and 1000 nm films is reduced compared with the detector with the 100 nm film. This would explain the decreased photocurrent as the thickness of the film increased. At the maximum applied bias voltage of 40 V, the photocurrents from the 100 nm, 500 nm, and 1000 nm films are 8.3 nA, 5.8 nA, and 2.2 nA, respectively. For reference, the photoconductivity of just the undoped Si substrate was obtained and, at 40 V bias voltage, the photocurrent measured was 0.08 nA. This confirms that the TiO_2_ films are photoconductive, while the Si substrate is not.

**Figure 6:**
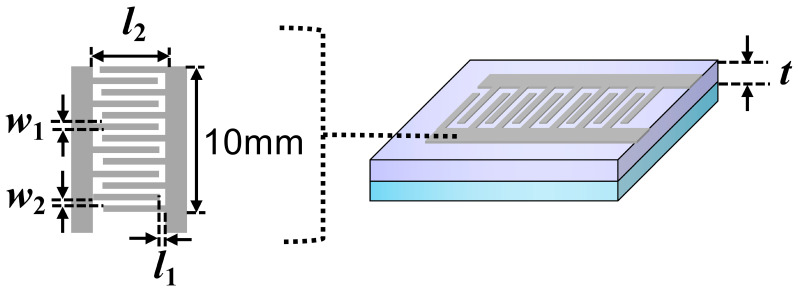
Dimensions of the aluminum electrode. *l*_1_ = 0.6 mm, *l*_2_ = 4.8 mm, $w_{1}$ = 0.4 mm, $w_{2}$ = 0.4 mm, and *n* = 13 interdigitated electrode arrays.

The photoconductivity of the TiO_2_ detector can be determined from the photocurrent and the structural dimensions of the electrodes as illustrated in Figure 6 as follows:

(4) $$\Delta i=J_{av}A$$

where Δi is the measured photocurrent, *A* is the cross-sectional area of the detector, and $J_{av}$ is the average current density given by

(5) $$J_{av}=\frac{J_{1}+J_{2}+\ldots +J_{n}}{n}$$

The current density *J* is related to the photoconductivity (*Z*) and electric field (*E*), as *J* = *ZE*. The electric field is further related to the bias voltage (*V*_bias_), the voltage *V*_2_ at the input of the board, and the distance between the electrode pair (*l*) according to $V_{\mathrm{bias}}-V_{2}=El$. The voltage *V*_2_ is very close to 0.7 V as the drop on the 100 ohm resistor is negligible due to the nanometric current flowing through it. The distance between the electrode pair is alternatingly equal to *l*_1_ or *l*_2_. Therefore, the average current density can be written as

(6) $$J_{av}=\frac{1}{2}Z\left[\frac{V_{\mathrm{bias}}-0.7}{l_{1}}+\frac{V_{\mathrm{bias}}-0.7}{l_{2}}\right]$$

Assuming that current is generated only between the electrode pairs, the cross-sectional area *A* can be calculated from

(7) $$A=\frac{n}{2}\left(tw_{1}\right)+\frac{n}{2}\left(tw_{2}\right)$$

where *t* is the thickness of the film, w is the width of the electrode, and *n* is the number of interdigitated electrode arrays. By combining Equations (5), (7) and (8), the photoconductivity is then given by

(8) $$Z=\frac{4\Delta i}{ntV_{\mathrm{bias}}\left(w_{1}+w_{2}\right)\left(\frac{1}{l_{1}}+\frac{1}{l_{2}}\right)}$$

The photoresistance of the detector is determined according to

(9) $$\Delta i=\frac{V_{\mathrm{bias}}-V_{2}\;}{R_{p}+\Delta \mathrm{photoresistance}}\;$$

where *R_p_* is the 100 Ω resistance of the potentiometer and *V*_2_ is the voltage at the detector’s bottom electrode, which is about 0.7 V. Because of the very small photocurrent, the voltage drop across the 100 Ω resistor will be much less than a millivolt. Hence, *V*_bias_ − 0.7 V is equal to the electric field times the distance between the electrodes and

(10) $$\Delta \mathrm{photoresistance}=\frac{El\;}{\Delta i}-R_{p}$$

**Figure 7:**
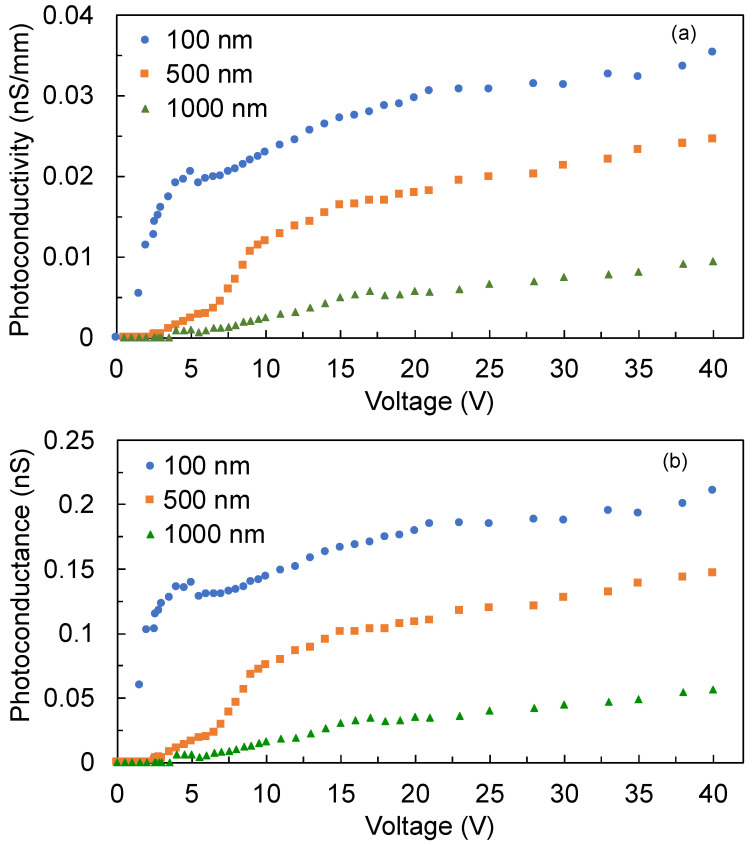
(**a**) Photoconductivity and (**b**) photoconductance of the detectors with 100 nm, 500 nm, and 1000 nm film thicknesses.

The photoconductance can be calculated as the inverse of the photoresistance. As shown in Figure 7a,b, the photoconductivity and photoconductance follow the observed trend in the photocurrent, whereby both decreased in the thicker films. In particular, the nonlinear behavior for low bias voltage, fewer than 10 V, is clearly observable.

**Figure 8:**
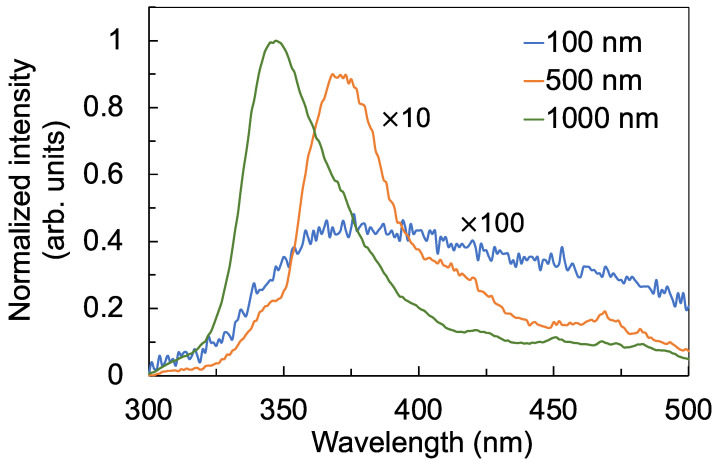
Photoluminescence emission spectra of the TiO_2_ thin films with thicknesses of 100 nm, 500 nm, and 1000 nm under 260 nm UV-C excitation. The luminescence intensity of the 500 nm and 100 nm films were zoomed-in by a factor of 10 and 100, respectively, for ease of comparison.

To further explore the nature of the defects and their role in decreasing the photocurrent, photoconductivity, and photoconductance, the photoluminescence emission from the films was evaluated when each of the films were excited by 260 nm UV-C photons. Figure 8 shows a broad luminescence from 325 nm to 450 nm. Comparing the luminescence spectra in Figure 8 with the transmission spectra in Figure 4a, the wavelength range of the broad luminescence coincides with the wavelength range where the transmittance decreased. Moreover, the luminescence intensity increased, while the transmittance decreased as the films became thicker. The luminescence emission can therefore be attributed to the radiative recombination of electrons in deep traps and holes in the valence band [30,33,34,35,36,37,38]. The increase in the luminescence intensity confirms that thicker films have more defect states. Although these defect states enhanced electron–hole recombination and therefore luminescence intensity, these defect states also served as electron traps that reduced the carrier mobility and carrier concentration, leading to the reduction in the photocurrent. These results suggest that although the 1000 nm film is more crystalline compared with the 100 nm film, as shown in Figure 3, the increased number of defects in thicker films has a more significant effect in decreasing the photoconductivity of the detector. A similar behavior was observed in ZnGa_2_O_4_ thin-film transistors, where the number of oxygen vacancies had a more pronounced effect on the transistor’s conductivity compared with the film’s crystallinity [37].

**Figure 9:**
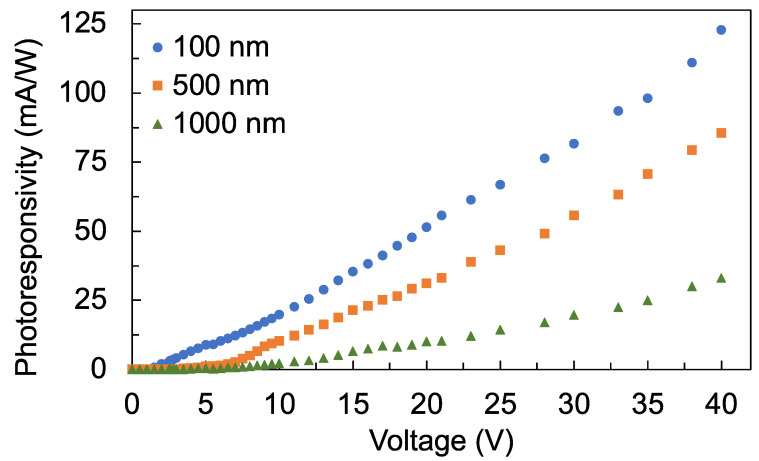
Photoresponsivity of the detectors with 100 nm, 500 nm, and 1000 nm film thicknesses.

Figure 9 shows the photoresponsivity ($R_{\lambda }$) of the detectors. The photoresponsivity was calculated using

(11) $$R_{\lambda }=\frac{I_{photo}}{P_{in}}$$

where $I_{photo}$ is the photocurrent and $P_{in}$ is the power of the 260 nm UV-C light incident on the detector, which is 18.7 μW. Since photoresponsivity is proportional to the photocurrent, the photoresponsivity of the detector also decreased as the films became thicker. At 40 V bias voltage, the photoresponsivities of the detectors with 100 nm, 500 nm, and 1000 nm film are 122.9 mA/W, 85.6 mA/W, and 33.1 mA/W, respectively. In comparison to previously reported UV-C photodetectors, the photoresponsivity of AlN film at 30 V bias voltage and 160 nm wavelength illumination is 6 mA/W [39]. The photoresponsivity of our TiO_2_ thin-film detector at 30 V bias voltage is 81.6 mA/W, 55.6 mA/W, and 19.6 mA/W for a film thickness of 100 nm, 500 nm, and 1000 nm, respectively. Previous work on TiO_2_ reported a photoresponsivity of 25.6 mA/W, which was measured directly from the TiO_2_ film with 253.65 nm excitation wavelength and 10 V bias voltage [40]. On the other hand, the photocurrent from our work was limited by the TIA chip. Other works reporting the photoconductivity of ZnO used longer excitation wavelengths which are in the UV-A region (315 nm to 400 nm). For instance, a photoresponsivity of 1 A/W with an excitation wavelength of 325 nm and bias voltages of 3 V [41] and 1.5 A/W with an excitation wavelength of 370 nm and bias voltage of 5 V [42] have been reported. As the excitation wavelength moves away from the band gap of the material, the photoresponsivity also tends to decrease. Therefore, the reported photoresponsivities from ZnO are higher compared with TiO_2_ or AlN.

**Figure 10:**
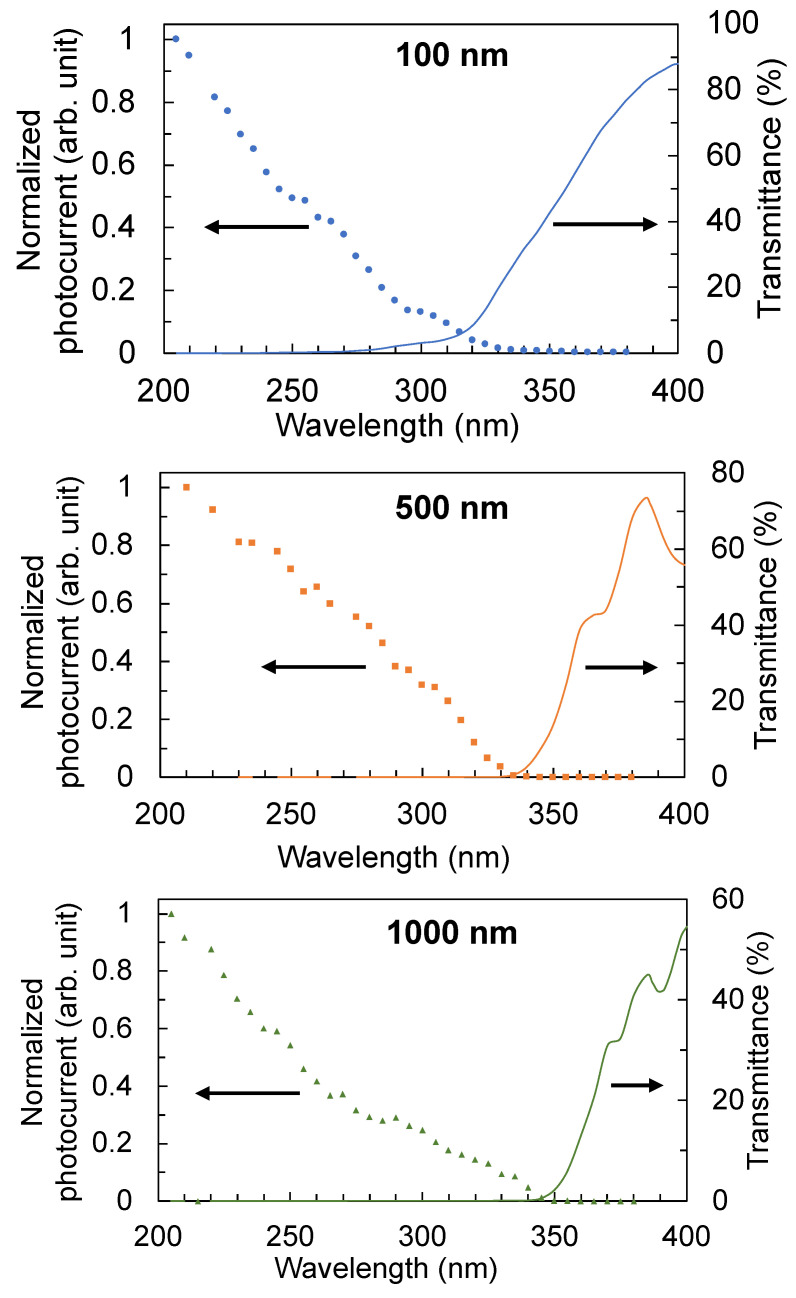
Wavelength response of the detectors with 100 nm, 500 nm, and 1000 nm film thicknesses. The cut-off wavelength is defined by the onset of a photocurrent being measured. The cut-off wavelength shifts to longer wavelengths as the thickness of the film increases, in accordance with the shift to longer wavelengths of the transmission and absorption edges. The photocurrents were corrected for the wavelength dependence of the excitation source.

The wavelength response of the TiO_2_ thin-film detectors measured at a fixed bias voltage of 40 V is shown in Figure 10. The intensity of the excitation source is not flat across the wavelengths considered (200 nm to 400 nm) because the monochromator efficiency and excitation lamp emissivity changes as a function of wavelength. Therefore, the measured photocurrent was normalized by the measured power of the excitation source for each wavelength considered. The cut-off wavelengths of the detectors with 100 nm, 500 nm, and 1000 nm film thicknesses are 320 nm, 330 nm, and 345 nm, respectively. The detector will not be responsive to wavelengths that are longer than the cut-off wavelength. The cut-off wavelength of the detector is observed to shift to longer wavelengths as the thickness of the film increases. This shift is consistent with the observed red shift in the transmission and absorption edges as well as the decrease in the band-gap energy as the film becomes thicker. These results suggest that the thickness of the film may be controlled to manipulate the wavelength response of the detectors to filter out unwanted longer wavelengths, especially if the trade-off in photoconductivity is acceptable for that specific application.

## 4. Conclusions

In conclusion, the development of a photoconductive detector in the UV-C wavelength region, particularly at 260 nm, using TiO_2_ thin films and a TIA, was explored. TiO_2_ films of 100 nm, 500 nm, and 1000 nm thicknesses were deposited on a high-resistivity undoped Si substrate by reactive DC magnetron sputtering. The photoresponsivities of the TiO_2_ thin-film detectors at 30 V bias voltage are 81.6 mA/W, 55.6 mA/W, and 19.6 mA/W for film thicknesses of 100 nm, 500 nm, and 1000 nm, respectively. These are higher than those of previously reported responsivity from AlN film, which was 6 mA/W. The effect of film thickness on the detectors’ photocurrent, photoconductivity, photoconductance, photoresponsivity, and wavelength response were investigated. Interestingly, the detector which used the thinnest 100 nm TiO_2_ film exhibited the best photoconductive characteristics. Although the thicker films had better crystallinity, they also had poorer optical quality, which was manifested by the decrease in their transmittance especially around the 300 nm to 450 nm wavelength region. The lower transmittance in the thicker films can be attributed to the increased defects. The increase in the number of defects in the thicker films was also confirmed by the photoluminescence emission spectra of the films, whereby defect-related photoluminescence appeared to increase in the thicker films. The wavelength response of the detector depends on the thickness of the film as their band gap shifted to lower energy as the film thickness increased. The thickness of the film could therefore be controlled to manipulate the wavelength response of the detectors to filter out unwanted longer wavelengths. Future development of a chip-based portable UV-C detector using TiO_2_ thin films will pave the way for new opportunities in a wide range of applications.

## Figures and Tables

**Table 1:** Strain, crystallite size, and crystallinity of the TiO_2_ films with 100 nm, 500 nm, and 1000 nm film thicknesses.

Thickness (nm)	Strain	Crystallite Size (nm)	Crystallinity (%)
100	2.4 × 10^−3^	9.6	50
500	4.5 × 10^−3^	23.5	78
1000	5.2 × 10^−3^	26.6	81

**Table 2:** Transmission edge and band-gap energy of the TiO_2_ films with 100 nm, 500 nm, and 1000 nm film thicknesses.

Thickness (nm)	Transmission Edge (nm)	Band Gap (eV)
100	320	3.49
500	335	3.44
1000	345	3.34

## Data Availability

The data presented in this study are available from the corresponding author upon reasonable request.
